# A systematic review and meta-analysis on sodium tanshinone IIA sulfonate injection for the adjunctive therapy of pulmonary heart disease

**DOI:** 10.1186/s12906-024-04434-0

**Published:** 2024-04-05

**Authors:** Huikai Shao, Fei Yu, Dongsheng Xu, Chunyan Fang, Rongsheng Tong, Lingguo Zhao

**Affiliations:** 1 Personalized Drug Therapy Key Laboratory of Sichuan Province, Sichuan Academy of Medical Sciences & Sichuan Provincial People’s Hospital, University of Electronic Science and Technology of China, Chengdu, 610072 China; 2 Sinopharm Dongfeng General Hospital, Hubei University of Medicine, 442008 Shiyan, China; 3 Institute of Pharmaceutical Analysis, College of Pharmacy, Jinan University, Guangzhou, 510006 China; 4 TCM Regulating Metabolic Diseases Key Laboratory of Sichuan Province, Hospital of Chengdu University of Traditional Chinese Medicine, Chengdu, 610072 China; 5 Center for Disease Prevention and Control of Baoan District, Shenzhen, 518101 China

**Keywords:** Adjunctive therapy, Sodium tanshinone IIA sulfonate injection, Pulmonary heart disease, Meta-analysis, Systematic review

## Abstract

**Aims:**

Sodium tanshinone IIA sulfonate (STS) injection has been widely used as adjunctive therapy for pulmonary heart disease (PHD) in China. Nevertheless, the efficacy of STS injection has not been systematically evaluated so far. Hence, the efficacy of STS injection as adjunctive therapy for PHD was explored in this study.

**Methods:**

Randomized controlled trials (RCTs) were screened from China Science and Technology Journal Database, China National Knowledge Infrastructure, Wanfang Database, PubMed, Sino-Med, Google Scholar, Medline, Chinese Biomedical Literature Database, Cochrane Library, Embase and Chinese Science Citation Database until 20 January 2024. Literature searching, data collection and quality assessment were independently performed by two investigators. The extracted data was analyzed with RevMan 5.4 and STATA 14.0. Basing on the methodological quality, dosage of STS injection, control group measures and intervention time, sensitivity analysis and subgroup analysis were performed.

**Results:**

19 RCTs with 1739 patients were included in this study. Results showed that as adjunctive therapy, STS injection combined with Western medicine showed better therapeutic efficacy than Western medicine alone for PHD by increasing the clinical effective rate (RR = 1.22; 95% CI, 1.17 to 1.27; *p* < 0.001), partial pressure of oxygen (MD = 10.16; 95% CI, 5.07 to 15.24; *p* < 0.001), left ventricular ejection fraction (MD = 8.66; 95% CI, 6.14 to 11.18; *p* < 0.001) and stroke volume (MD = 13.10; 95% CI, 11.83 to 14.38; *p* < 0.001), meanwhile decreasing the low shear blood viscosity (MD = -1.16; 95% CI, -1.57 to -0.74; *p* < 0.001), high shear blood viscosity (MD = -0.64; 95% CI, -0.86 to -0.42; *p* < 0.001), plasma viscosity (MD = -0.23; 95% CI, -0.30 to -0.17; *p* < 0.001), hematokrit (MD = -8.52; 95% CI, -11.06 to -5.98; *p* < 0.001), fibrinogen (MD = -0.62; 95% CI, -0.87 to -0.37; *p* < 0.001) and partial pressure of carbon dioxide (MD = -8.56; 95% CI, -12.09 to -5.02; *p* < 0.001).

**Conclusion:**

STS injection as adjunctive therapy seemed to be more effective than Western medicine alone for PHD. However, due to low quality of the included RCTs, more well-designed RCTs were necessary to verify the efficacy of STS injection.

**Supplementary Information:**

The online version contains supplementary material available at 10.1186/s12906-024-04434-0.

## Introduction

Pulmonary heart disease (PHD) is considered to be caused by lesions in lung and bronchial tissue, as well as the pulmonary vascular system [1]. It was reported that PHD accounted for 10–30% of all heart failure patients and over 40% of chronic lung disease patients had the signs of PHD at autopsy in United states [2, 3]. Nowadays, many Western medicines have been successfully employed to treat PHD, including vasodilators, antibiotics, diuretics, etc. However, the clinical efficacy of these Western medicines is usually unsatisfactory [4]. Recently, a large number of researches showed that traditional Chinese medicine (TCM) had positive therapeutic effects in the treatment of PHD [5–10].

Sodium tanshinone IIA sulfonate (STS) injection is the water-soluble derivative of tanshinone IIA and the chemical structure is shown in **Fig ****S1**[11]. STS is a pharmacologically active compound extracted from traditional Chinese medicine named as Danshen [12, 13]. STS injection is usually diluted with 5% glucose injection of 250 or 500 mL and then given to the PHD patients through intravenous drip (once a day). It was reported that STS injection also showed great potential in treating stroke, pulmonary diseases, hepatic diseases and sepsis [11]. Recent studies showed that STS injection could inhibit proliferation of pulmonary smooth muscle cells and stimulate expression of Kv2.1 [14], as well as suppress the expression of canonical transient receptor pulmonary arterial smooth muscle cells derived from the rat model of pulmonary hypertension [15]. Moreover, STS injection could inhibit hypoxia-induced enhancement of store-operated calcium entry (SOCE) in pulmonary arterial smooth muscle cells *via* activation of the PKG-PPAR-γ signaling axis [13].

Currently, a great deal of studies has reported that STS injection combined with Western medicine has great potentials in the treatment of PHD with positive results. As far as we know, neither systematic review nor meta-analysis about the efficacy of STS injection as adjunctive therapy for treating PHD has yet been reported. Hence, the aim of this study is to summarize the available evidence from the published randomized controlled trials (RCTs) to demonstrate the efficacy of STS injection as adjunctive therapy for PHD with a comprehensive PRISMA-compliant systematic review and meta-analysis [16–21].

## Methods

### Inclusion criteria

Two investigators (H.K. Shao and D.S. Xu) independently searched and screened the eligible RCTs according to the PICOS principle: (1) Patients (P): The study population was the PHD patients which met with the diagnostic criteria for chronic PHD of China or the World Health Organization (WHO) diagnostic criteria for PHD. (2) Intervention (I): STS injection combined with Western medicine was given to the PHD patients in the treatment group. (3) Comparisons (C): Western medicine alone was given to the PHD patients in the control group. (4) Outcomes (O): clinical effective rate was selected as the primary outcome in this study; partial pressure of oxygen (PaO_2_), left ventricular ejection fraction (LVEF), stroke volume (SV), low shear blood viscosity (LBV), plasma viscosity (PV), high shear blood viscosity (HBV), hematokrit (HCT), fibrinogen (FIB), partial pressure of carbon dioxide (PaCO_2_) and adverse reactions were chosen as the secondary outcomes. (5) Study design (S): the included studies should be randomized controlled trials (RCTs).

### Exclusion criteria

RCTs should be removed from this meta-analysis when they could not satisfy the above inclusion criteria and: (1) In the treatment or control group, the formulation and dosages of interventions were not provided; (2) Incomplete data in the RCTs or repeated publications were found; (3) Other Chinese herb medicine was used in the RCT; (4) Type of articles was meeting abstracts, case reports and literature reviews; (5) Obvious data obfuscations and wrong data were found.

### Information sources

To screen the eligible RCTs, many databases including Google Scholar, China National Knowledge Infrastructure (CNKI), PubMed, Medline, Wanfang Database, China Science and Technology Journal Database (VIP), Embase, Sino-Med, Chinese Biomedical Literature Database, Cochrane Library and Chinese Science Citation Database were systematically searched. The nearest update for the RCTs was performed on 20 January 2024.

### Search strategies

To select the eligible RCTs, the following keywords in English databases were searched and scanned by joint or separate method: STS injection, STS, PHD and *cor pulmonale*. The following keywords in Chinese databases were searched and scanned by joint or separate method as follows: Danshentong IIA, Danshentong IIA Huangsuanna Zhusheye [STS injection], Danshentong IIA huangsuanna [Sodium tanshinone IIA sulfonate], Feixinbing [PHD or *cor pulmonale*] and Feiyuanxingxinzangbing [PHD or *cor pulmonale*]. In addition, the references in the included RCTs were read to obtain the eligible RCTs.

### Study selection

To obtain the included RCTs, the above databases were systematic searched by two investigators (H.K. Shao and D.S. Xu) according to the inclusion and exclusion criteria. Any dispute in the process of study selection was solved by discussion with a third investigator (L.G. Zhao).

### Data collection process

Full text of the included RCTs was carefully read by one investigator (H.K. Shao) and the relevant data was extracted at the same time. Another investigator (D.S. Xu) verified the completeness and accuracy of the collected data. Any disputes during the process of data collection were solved by discussion with a third investigator (L.G. Zhao). To analyze the extracted data, the software STATA 14.0 (Stata Corp LLC, Texas, USA) and Review Manager 5.4 (Cochrane Collaboration, Nordic Cochrane center, Copenhagen, Denmark) were employed in this meta-analysis.

### Data items

One investigator (H.K. Shao) collected the following data items from the included RCTs: (a) First author’s name; (b) Sample size of the patients; (c) Publication date; (d) Interventions in the treatment group and control group; (e) Outcome measures; (f) Duration of interventions; (g) Adverse reactions. The accuracy and completeness of data items were carefully checked by another investigator (D.S. Xu).

### Quality assessment

Methodological qualities of the included RCT were evaluated with Jadad scoring system and it was ranged from 0 to 5 scores (Table S1) [17–22]. According to the items of randomization, withdrawals (dropouts) and blinding, the methodological qualities were independently assessed by two investigators (H.K. Shao and D.S. Xu). Scores were given to the included RCTs if they meet with the following criteria: Method of randomization was provided, 1 score; suitable randomization method was given, 1 score; withdrawals and dropouts were given, 1 score; double blinding method was given, 1 score; suitable double-blinding method was given, 1 score. RCT obtained 1 or 2 scores was considered as the low-quality study and 3 to 5 scores was considered as high-quality study [17–21]. A third investigator (L.G. Zhao) was involved to solve the disputes in the process of quality assessment. The risk of bias was independently evaluated by two investigators using the Cochrane risk of bias (ROB) tool. Seven aspects including random sequence generation, allocation concealment, blinding of participants and personnel, blinding of outcome assessment, incomplete outcome data, selective reporting and other bias were included in the ROB tool and each aspect was divided into “low risk”,“high risk” and “unclear risk”. Disagreements in the evaluation of the risk of bias were resolved by discussions with the third investigator (L.G. Zhao) [23].

### Statistical analysis

Statistical analysis in this meta-analysis was conducted using the software RevMan 5.4. Dichotomous variables were expressed as the pooled risk ratios (RR) with 95% confidence interval (CI), whereas continuous variables were expressed as the weighted mean difference (MD) with 95% CI. To assess the heterogeneity among the RCTs, Chi-squared test and I^2^ statistic were used. If I^2^ > 50% or *p* < 0.05, it indicated that there was significant heterogeneity and we should use random-effect model to assess the data. If not, the fixed-effect model should be chosen. Z-test was adopted to estimate whether the overall effect of STS injection combined with Western medicine was better than Western medicine alone for PHD. It suggested that significant statistical difference was observed when *p* < 0.05 [23, 24]. Meta-regression was performed to investigate whether the potential presence of effect modifiers explained any of the heterogeneity of treatment effects of STS injection between RCTs. In this study, meta-regression was conducted basing on the following variables: STS injection dosage, control group measures and intervention time. Significant differences were considered if *p* < 0.05.

### Sensitivity and subgroup analysis

Sensitivity analysis was performed to estimate the impact of the included RCTs with low-quality on the overall effects of STS injection for PHD basing on the Jadad scoring system. To assess if the overall effect was homogeneous within subgroups, subgroup analysis was performed basing on the dosage of STS injection (the subgroups with specific dosage and a dynamic range dosage), control group measures (the subgroups with only enalapril and other medicines) and intervention time.

### Risk of bias across studies

To obtain accuracy results for the publication bias, Egger’s test and Begg’s test were also conducted.

## Results

### Study selection process

**Fig. 1:**
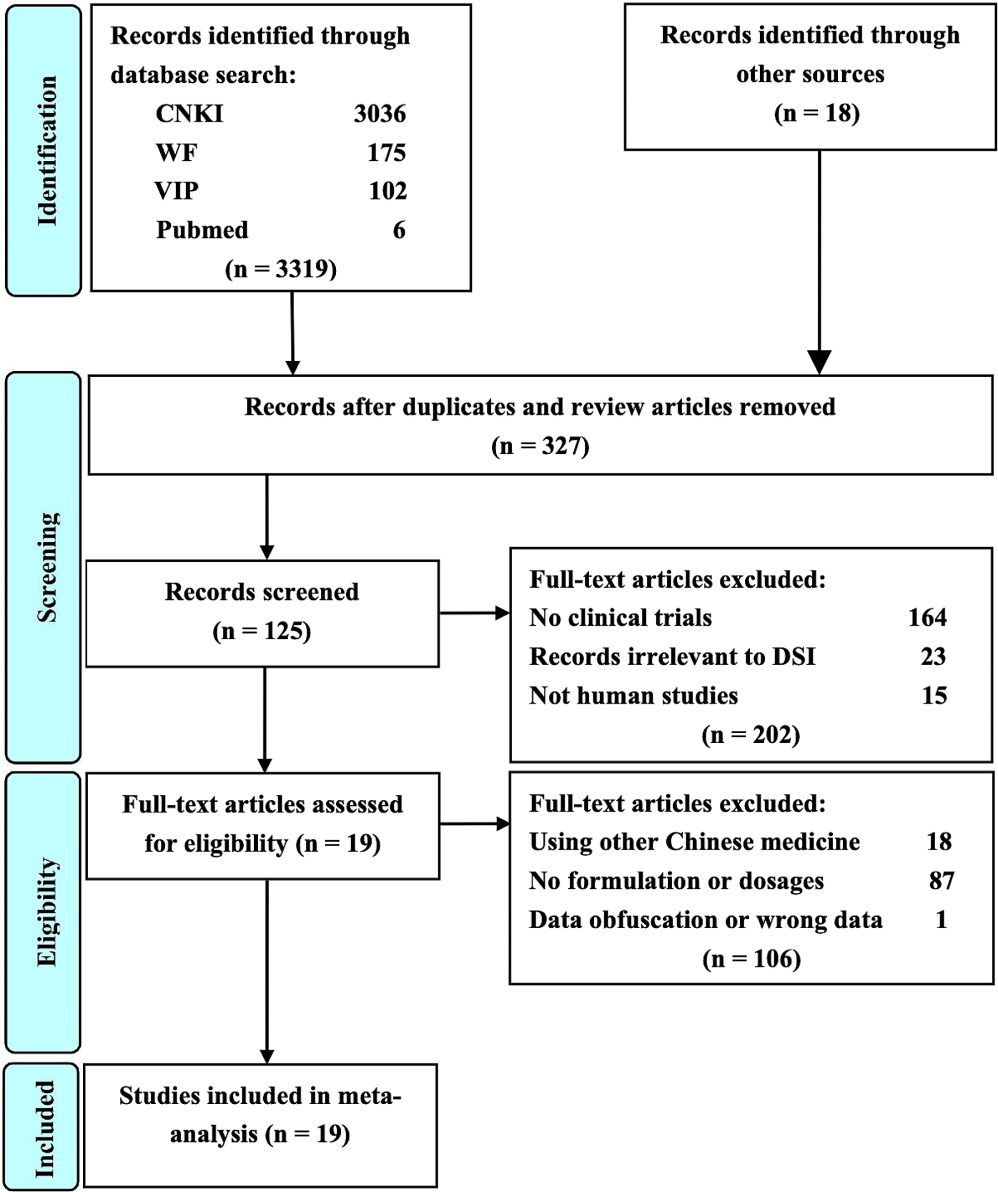
PRISMA flow diagram of study selection process. CNKI is China National Knowledge Infrastructure; VIP is Chinese Scientific Journal Database; WF is Wangfang Data; Other sources are Google Scholar, Medline, Sino-Med, Chinese Biomedical Literature Database, Chinese Science Citation Database, Cochrane Library and Embase

As shown in Fig. 1, the study selection process was described. At the beginning, 3337 potential studies were identified from the databases. Due to the duplications, animal experiments and literature reviews, 3010 studies were excluded according to the eligible criteria. Full texts of 327 studies were downloaded and carefully read basing on the inclusion criteria. Finally, a total of 19 RCTs were chosen for the methodological quality evaluation and further meta-analysis.

### Study characteristics

Table 1 presented the features of the included 19 RCTs. All the RCTs were reported on Chinese journals and the date of publication ranged from 2011 to 2021 [25–43]. The sample size of the PHD patients was from 44 to 146 with 1739 patients in all and the average value was 91.53. Duration of intervention for treating PHD was 10 days at least, 14 days mostly. For the control group, several kinds of conventional Western medicine were given, including phentolamine mesylate injection, sodium nitroprusside, enalapril, meglumine adensine cyclphsphate injection, aminophylline, salbutamol, roxithromycin, ceftazidime, alprostadil and sildenafil. Basing on the control group, different dosage of STS injection from 40 to 80 mg (once a day) were employed in the treatment group. Among the included 19 RCTs, clinical effective rate was chosen as the therapeutic indicator in 18 RCTs. There were 6 RCTs that reported LBV and/or HBV as the outcomes. PV was chosen as the outcome by 12 RCTs. There were 8 RCTs reported HCT as their outcome measure. FIB was chosen as the outcome measure by 7 RCTs. The outcome measure PaO_2_ was selected by 5 RCTs and LVEF was reported by 6 RCTs. SV was elected as outcome measure by 5 RCTs. Moreover, there were 4 RCTs that reported PaCO_2_ as their outcome measures.

**Table 1:** Characteristics of RCTs on STS injection for PHD

Study	Country	Year	Intervention		Sample	Follow-up (day)	Outcome measures
			Treatment group	Control group			
Chai YQ	China	2017	STS injection 50 mg/d, Phentolamine mesylate injection 20 mg/d	Phentolamine mesylate injection 20 mg/d	78	14	①②③④⑤⑥⑦⑧⑨⑩
Cheng KF	China	2017	STS injection 80 mg/d, sodium nitroprusside 10 mg/d	Sodium nitroprusside 10 mg/d	120	14	①
Chen JX	China	2016	STS injection 50 mg/d, enalapril 24 ~ 32 mg/d	Enalapril 24 ~ 32 mg/d	96	14	①④⑤
Chen SX	China	2015	STS injection 40 ~ 80 mg/d, enalapril 10 ~ 40 mg/d	Enalapril 10 ~ 40 mg/d	100	14	①④⑤⑥⑨⑩
Liang ZC	China	2016	STS injection 40 ~ 80 mg/d, enalapril 20 ~ 40 mg/d	Enalapril 20 ~ 40 mg/d	84	14	①②③④⑤⑥ ⑨⑩
Lin JY	China	2016	STS injection 80 mg/d, enalapril 20 mg/d	Enalapril 20 mg/d	100	14	①②③④⑤⑥
Li SY	China	2014	STS injection 40 ~ 60 mg/d, enalapril 20 ~ 32 mg/d	Enalapril 20 ~ 32 mg/d	146	14	①④⑤
Liu JL	China	2017	STS injection 40 ~ 80 mg/d, enalapril 5 ~ 20 mg/d	Enalapril 5 ~ 20 mg/d	70	28	①
Lu CB	China	2014	STS injection 50 mg/d, enalapril 20 ~ 30 mg/d	Enalapril 20 ~ 30 mg/d	62	28	①
Lu P	China	2017	STS injection 60 mg/d, meglumine adensine cyclphsphate injection 180 mg/d	Meglumine adensine cyclphsphate injection 180 mg/d	60	10	①②③⑦
Ma Y	China	2015	STS injection 40 ~ 60 mg/d, enalapril 20 ~ 32 mg/d	Enalapril 20 ~ 32 mg/d	78	14	①④⑤
Mei WH	China	2018	STS injection 60 mg/d, enalapril 20 mg/d	Enalapril 20 mg/d	70	28	①
Ni F	China	2018	STS injection 50 mg/d, phentolamine mesylate injection 10 mg/d	Phentolamine mesylate injection 10 mg/d	109	15	①②③④⑥⑦⑧
Song HH	China	2017	STS injection 50 mg/d, enalapril 20 ~ 40 mg/d	Enalapril 20 ~ 40 mg/d	44	28	①④⑤
Wang W	China	2021	STS injection 40 ~ 80 mg/d, enalapril 10 ~ 20 mg/d	Enalapril 10 ~ 20 mg/d	110	14	①②③④⑥
Wang XQ	China	2019	STS injection 40 ~ 80 mg/d, enalapril 5 ~ 20 mg/d	Enalapril 5 ~ 20 mg/d	100	28	①⑨⑩
Wang XR	China	2011	STS injection 80 mg/d, aminophylline 0.5 g, salbutamol 12 mg, roxithromycin 600 mg, ceftazidime 2 g	Aminophylline 0.5 g, salbutamol 12 mg, roxithromycin 600 mg, ceftazidime 2 g	120	14	④⑥
Yang YQ	China	2012	STS injection 40 ~ 80 mg/d, enalapril 5 ~ 20 mg/d	Enalapril 5 ~ 20 mg/d	114	28	①④⑦⑧⑨⑩
Zhang J	China	2018	STS injection 40 ~ 80 mg/d, alprostadil 1 ~ 2 mL/d, sildenafil 50 mg/d	Alprostadil 1 ~ 2 mL/d, sildenafil 50 mg/d	78	14	①⑦⑧⑨

① Clinical effective rate; ② LBV; ③ HBV; ④ PV; ⑤ HCT; ⑥ FIB; ⑦ PaO_2_; ⑧ PaCO_2_; ⑨ LVEF; ⑩ SV

### Risk of bias in individual studies

In this study, Jadad score was employed to estimate the methodological quality. As described in Tables 2 and 8 RCTs obtained 3 scores, 7 RCTs acquired 2 scores, the rest of 4 RCTs acquired 1 score, indicating that there were 8 high-quality RCTs and 11 low-quality RCTs.

**Table 2:** Quality of the included RCTs

Study	J1	J2	J3	J Score
Chai YQ, 2017	1	0	1	2
Cheng KF, 2017	1	0	1	2
Chen JX, 2016	0	0	1	1
Chen SX, 2015	2	0	1	3
Liang ZC, 2016	2	0	1	3
Lin JY, 2016	2	0	1	3
Li SY, 2014	1	0	1	2
Liu JL, 2017	1	0	1	2
Lu CB, 2014	0	0	1	1
Lu P, 2017	2	0	1	3
Ma Y, 2015	1	0	1	2
Mei WH, 2018	2	0	1	3
Ni F, 2018	1	0	1	2
Song HH, 2017	0	0	1	1
Wang W, 2021	1	0	1	2
Wang XQ, 2019	2	0	1	3
Wang XR, 2011	2	0	1	3
Yang YQ, 2012	2	0	1	3
Zhang J, 2018	0	0	1	1

### Clinical effective rate

**Fig. 2:**
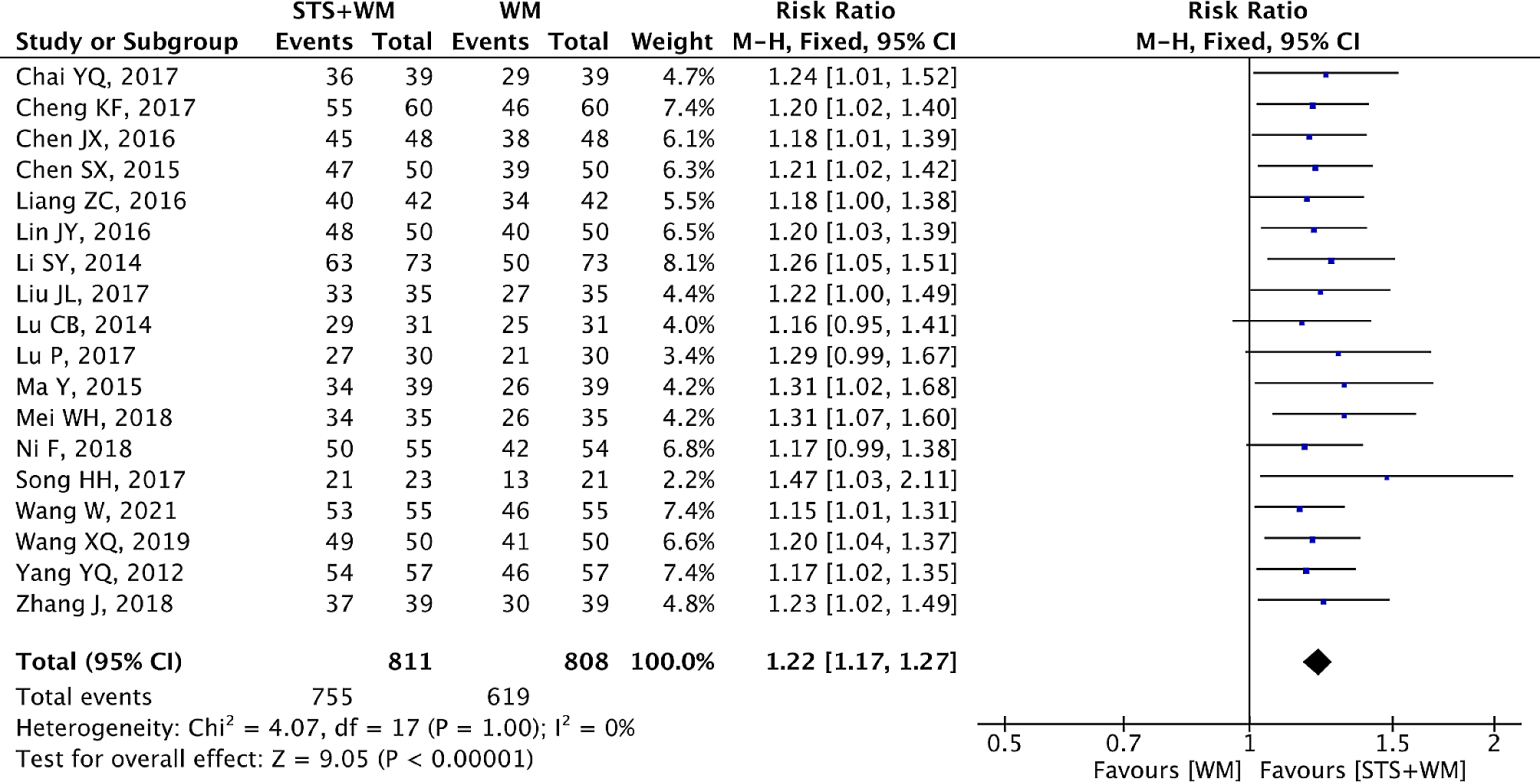
Forest plot of the clinical effective rate of STS injection as adjunctive therapy vs. Western medicine alone for PHD. STS is sodium tanshinone IIA sulfonate injection and WM is Western medicine

Clinical effective rate was chosen as the outcome by 18 RCTs. The clinical effective rate referred to the ratio of the responders to total PHD patients. The definition of responder was based on efficacy criteria of the Chinese National Conference on *Cor* Pulmonale in the years of 1977/1980 and an improvement of at least one class in the cardiopulmonary function was defined as the efficacy criteria. The calculation formula was defined as follows: (the number of remarkable recovery patients + the number of improved recovery patients)/total number of PHD patients × 100%. The efficacy was divided into three levels: ineffective, improved recovery and remarkable recovery. If the clinical symptoms were completely ameliorated and the cardiac function was improved by two levels, it could be considered as a remarkable recovery. For improved recovery, relief from clinical symptoms and an improvement in cardiac function by one level appeared in PHD patients. As depicted in Fig. 2, since there was no heterogeneity (*p* = 1.00, I^2^ = 0%), the fixed-effect model was used to analyze the collected data. The pooled RR value was 1.22 (95% CI, 1.17 to 1.27; Z = 9.05, *p* < 0.001), indicating that as an adjunctive therapy, STS injection combined with Western medicine had better therapeutic efficacy in treating PHD than Western medicine alone. To provide an accuracy result for publication bias, Begg’s test and Egger’s test were introduced. Results from Begg’s test (Z = 3.26, *p* = 0.001) and Egger’s test (t = 6.18, *p* = 0) also suggested that there was publication bias. Sensitivity analysis was performed to assess the influence of low-quality RCTs on the overall effects of STS injection combined with Western medicine. No significant difference was detected when the lower-quality RCTs were gradually excluded as shown in Table 3. There were little changes (0.01 in magnitude) between the RCTs with high-quality (Jadad scores ≥ 3) and that with low-quality (Jadad scores < 3). Subgroup analysis based on STS injection dosage (the subgroups with specific dosage and a dynamic range dosage), control group measures (the subgroups with only enalapril and other medicines) and intervention time (the subgroups with intervention time less than or more than 14 days) indicated that there was no difference between these factors, consistently demonstrating that STS injection could improve the efficacy for PHD.

**Table 3:** Sensitivity and subgroups analysis based on clinical effective rate

	Group	No. of RCTs	No. of patients	RR	95% CI	Z	P(effect)	I^2^	χ^2^	P(het)
Jadad score	0–5	18	1619	1.22	1.17, 1.27	9.05	< 0.00001	0%	4.07	1.00
	1–5	18	1619	1.22	1.17, 1.27	9.05	< 0.00001	0%	4.07	1.00
	2–5	14	1339	1.21	1.16, 1.27	8.14	< 0.00001	0%	2.54	1.00
	3–5	7	628	1.21	1.14, 1.29	5.98	< 0.00001	0%	1.11	0.98
	< 3	11	991	1.22	1.15, 1.29	6.84	< 0.00001	0%	2.98	0.98
	≥ 3	7	628	1.21	1.14, 1.29	5.98	< 0.00001	0%	1.11	0.98
Dosage of STS injection	Specific dosage	9	739	1.22	1.15, 1.30	6.19	< 0.00001	0%	2.48	0.96
	Dynamic range	9	880	1.21	1.14, 1.28	6.60	< 0.00001	0%	1.49	0.99
Control group measures	Using enalapril	13	1174	1.22	1.16, 1.28	7.84	< 0.00001	0%	3.59	0.99
	Other medicines	5	445	1.21	1.12, 1.32	4.54	< 0.00001	0%	0.49	0.97
Intervention time	≤ 14 d	11	1050	1.22	1.15, 1.28	7.13	< 0.00001	0%	1.72	1.00
	> 14 d	7	569	1.22	1.14, 1.30	5.59	< 0.00001	0%	2.35	0.89

### LBV

**Fig. 3:**
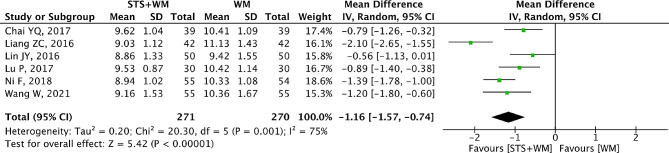
Forest plot of LBV on STS injection as adjunctive therapy vs. Western medicine alone for PHD. STS is sodium tanshinone IIA sulfonate injection and WM is Western medicine

As shown in Fig. 3, six RCTs reported LBV as their outcome measure. Owing to the high heterogeneity (*p* = 0.001, I^2^ = 75%) among the six RCTs, the random effect model was selected. In comparison with Western medicine alone, the results indicated that STS injection as an adjunctive therapy can decrease the LBV (MD = -1.16; 95% CI, -1.57 to -0.74; *p* < 0.001). As shown in Table S2, sensitivity analysis demonstrated that the results were robust. Due to the large heterogeneity in the included studies, subgroup analysis was performed basing on STS injection dosage, control group measures and intervention time. However, results from subgroup analysis showed that these parameters had no significant interaction. In addition, Egger’s test (t = 0.21, *p* = 0.847) and Begg’s test (Z = 0, *p* = 1.00) were performed and the results indicated that there was no publication bias among the six RCTs.

### HBV

**Fig. 4:**
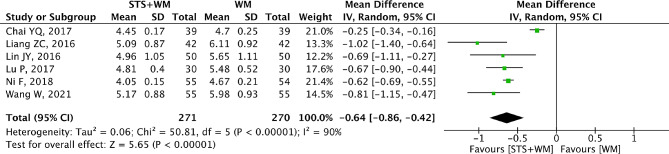
Forest plot of HBV on STS injection as adjunctive therapy vs. Western medicine alone for PHD. STS is sodium tanshinone IIA sulfonate injection and WM is Western medicine

The outcome HBV was selected as the therapeutic indicator by six RCTs. The random effect model was selected because of significant heterogeneity (*p* < 0.001, I^2^ = 90%). As shown in Fig. 4, STS injection as adjunctive therapy had more power in decreasing the level of HBV comparing with Western medicine alone (MD = -0.64; 95% CI, -0.86 to -0.42; *p* < 0.001). As shown in Table S3, sensitivity analysis demonstrated that the results were robust. Due to the large heterogeneity in the included studies, subgroup analysis was performed basing on STS injection dosage, control group measures and intervention time. However, results from subgroup analysis showed that these parameters had no significant interaction. To evaluate the publication bias, Egger’s test (t = -0.68, *p* = 0.533) and Begg’s test (Z = 0, *p* = 1.00) were performed and results indicated that there was no publication bias within the six RCTs.

### PV

**Fig. 5:**
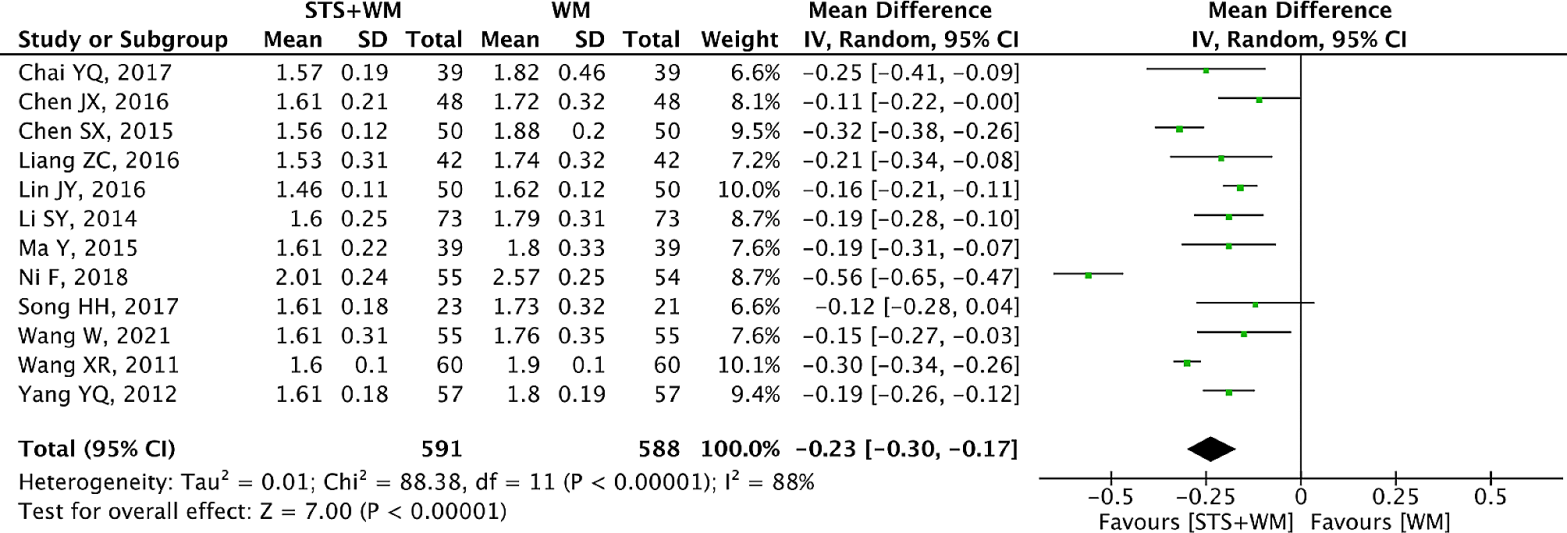
Forest plot of PV on STS injection as adjunctive therapy vs. Western medicine alone for PHD. STS is sodium tanshinone IIA sulfonate injection and WM is Western medicine

The outcome PV was reported by twelve RCTs and the results were expressed in Fig. 5. Since significant heterogeneity (*p* < 0.001, I^2^ = 88%) was found, the random effect model was selected for analyzing the collected data. Compared with Western medicine alone, the adjunctive use of STS injection seemed more effective in decreasing PV (MD = -0.23; 95% CI, -0.30 to -0.17; *p* < 0.001). As shown in Table S4, robust results were found in the process of sensitivity analysis. Owing to the large heterogeneity within the included studies, subgroup analysis was conducted basing on STS injection dosage, control group measures and intervention time. Results from subgroup analysis showed that these parameters had no significant interaction. Moreover, results from Egger’s test (t = 0.53, *p* = 0.606) and Begg’s test (Z = 0.21, *p* = 0.837) indicated that no publication bias was observed among the thirteen RCTs.

### HCT

**Fig. 6:**
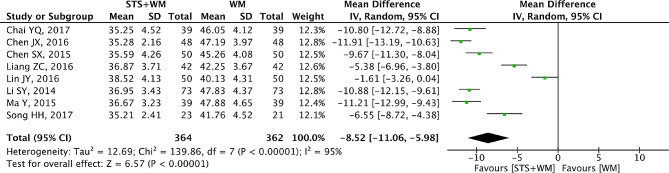
Forest plot of HCT on STS injection as adjunctive therapy vs. Western medicine alone for PHD. STS is sodium tanshinone IIA sulfonate injection and WM is Western medicine

There were 8 RCTs involving 726 patients that reported HCT as the outcome measure. In Fig. 6, we could find that random effect model was used to analyze the data because of significant heterogeneity (*p* < 0.001, I^2^ = 95%). This result demonstrated that STS injection as adjunctive therapy had better effects in decreasing HCT comparing with using Western medicine alone (MD = -8.52; 95% CI, -11.06 to -5.98; *p* < 0.001). As shown in Table S5, robust results were found in the process of sensitivity analysis. Owing to the large heterogeneity within the included studies, subgroup analysis was conducted basing on STS injection dosage, control group measures and intervention time. Results from subgroup analysis showed that these parameters had no significant interaction. To prove if there was potential publication bias, Egger’s test (t = 0.98, *p* = 0.364) and Begg’s test (Z = 0.87, *p* = 0.386) were performed and results indicated that there was no publication bias among the eight RCTs.

### FIB

**Fig. 7:**
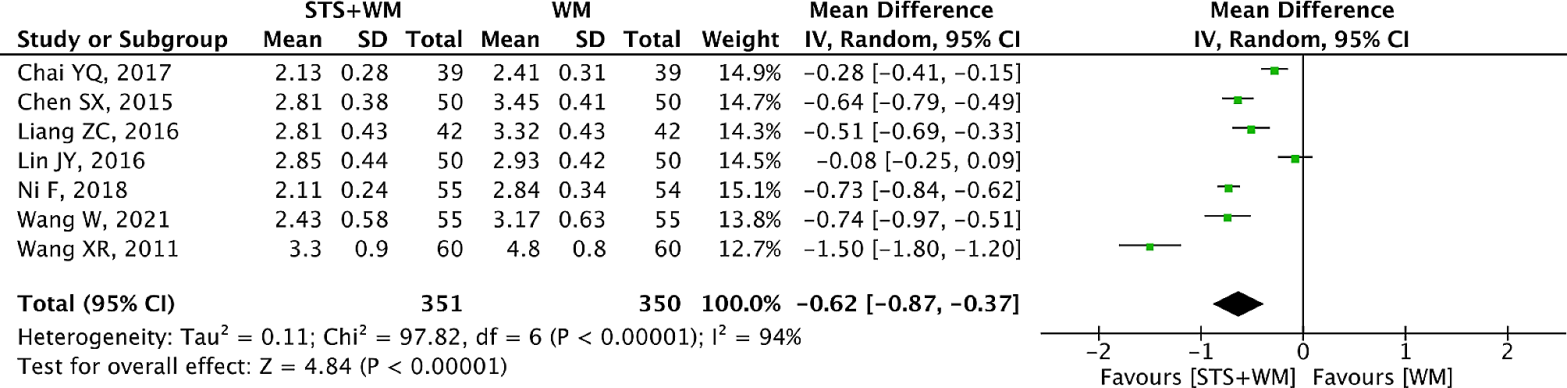
Forest plot of FIB on STS injection as adjunctive therapy vs. Western medicine alone for PHD. STS is sodium tanshinone IIA sulfonate injection and WM is Western medicine

FIB was reported in seven RCTs. There was high heterogeneity among these eight RCTs (*p* < 0.001, I^2^ = 94%) and the random effect model was conducted in this meta-analysis. As shown in Fig. 7, the overall MD of FIB was − 0.62 (95% CI, -0.87 to -0.37; *p* < 0.001) among the eight RCTs, indicating that STS injection combined with Western medicine was more effective in decreasing FIB than Western medicine alone. In Table S6, results from sensitivity analysis were robust. Owing to the large heterogeneity within the included studies, subgroup analysis was conducted basing on STS injection dosage, control group measures and intervention time. Results from subgroup analysis showed that these parameters had no significant interaction. In addition, Egger’s test (t = -0.79, *p* = 0.467) and Begg’s test (Z = 0.60, *p* = 0.548) were carried out to detect the potential publication bias and results indicated that there was no publication bias.

### PaO_2_

**Fig. 8:**
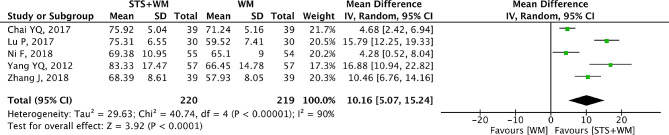
Forest plot of PaO_2_ on STS injection as adjunctive therapy vs. Western medicine alone for PHD. STS is sodium tanshinone IIA sulfonate injection and WM is Western medicine

PaO_2_ was selected as the outcome measure by five RCTs. The random effect model was adopted since a high heterogeneity within the 5 RCTs (*p* < 0.001, I^2^ = 90%) was detected. As shown in Fig. 8, the overall MD for PaO_2_ was 10.16 (95% CI, 5.07 to 15.24; *p* < 0.001) within the included 5 RCTs, which demonstrated that STS injection as adjunctive therapy presented better effect in increasing the level of PaO_2_ than Western medicine alone. Table S7 indicated that results of sensitivity analysis were robust. Owing to the large heterogeneity within the included studies, subgroup analysis was conducted basing on STS injection dosage, control group measures and intervention time. Results from subgroup analysis showed that the large heterogeneity could be attributed to the inconsistency of control group measures in the included studies. In the 5 RCTs, only enalapril was used for control group measures in one RCTs and the rest of four RCTs selected other medicines including phentolamine mesylate injection, meglumine adensine cyclphsphate injection, alprostadil and sildenafil. Different control group measures might cause the large heterogeneity. To estimate the publication bias for the 5 included RCTs, Egger’s test (t = 1.59, *p* = 0.21) and Begg’s test (Z = 0.24, *p* = 0.806) were employed in this study. According to the results, no publication bias was found.

### PaCO_2_

**Fig. 9:**
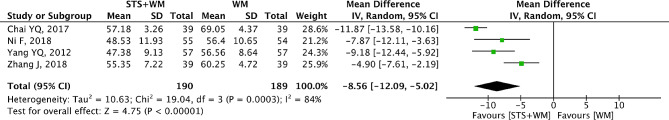
Forest plot of PaCO_2_ on STS injection as adjunctive therapy vs. Western medicine alone for PHD. STS is sodium tanshinone IIA sulfonate injection and WM is Western medicine

Four RCTs with 379 patients provided data for PaCO_2_. As shown in Fig. 9, STS injection combined with Western medicine could significantly reduce PaCO_2_ for the PHD patients (MD = -8.56; 95% CI, -12.09 to -5.02; *p* < 0.001). In this meta-analysis, a random effect model was used since there was a high heterogeneity (*p* = 0.0003, I^2^ = 84%) among these four RCTs. Table S8 indicated that results of sensitivity analysis were robust. Owing to the large heterogeneity within the included studies, subgroup analysis was conducted basing on STS injection dosage, control group measures and intervention time. However, results from subgroup analysis showed that the large heterogeneity could be attributed to the inconsistency of STS injection dosage in the included studies. Publication bias for the included RCTs was evaluated by STATA 14.0 with Begg’s test and Egger’s test. Evidence from Egger’s test (t = 1.19, *p* = 0.357) and Begg’s test (Z = 0.34, *p* = 0.734) proved that no publication bias was detected.

### LVEF

**Fig. 10:**
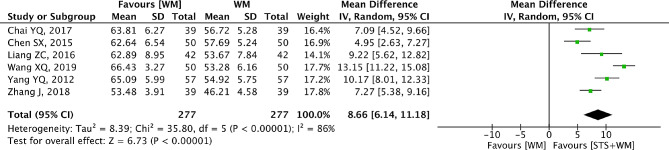
Forest plot of LVEF on STS injection as adjunctive therapy vs. Western medicine alone for PHD. STS is sodium tanshinone IIA sulfonate injection and WM is Western medicine

Six RCTs provided the therapeutic indicator LVEF as the outcome measure. In Fig. 10, we could find that a significant heterogeneity (*p* < 0.001, I^2^ = 86%) emerged and the random effect model was adopted. Moreover, it was obvious to find that the efficacy of STS injection as adjunctive therapy was more effective in increasing LVEF for the PHD patients than Western medicine alone (MD = 8.66; 95% CI, 6.14 to 11.18; *p* < 0.001). In Table S9, we could observe that there was big difference between the low-quality (Jadad score < 3) and high-quality (Jadad score ≥ 3) RCTs from results of sensitivity analysis. Besides, subgroup analysis basing on STS injection dosage, control group measures and intervention time was performed and results showed that the large heterogeneity could be attributed to the inconsistency of STS injection dosage, control group measures and intervention time among the included studies. Hence, publication bias among the six RCTs was evaluated with Egger’s test and Begg’s test. The evidence from Egger’s test (t = -0.47, *p* = 0.66) and Begg’s test (Z = 0, *p* = 1.00) showed that publication bias might exist within the seven RCTs.

### SV

**Fig. 11:**
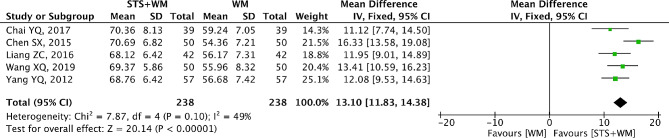
Forest plot of SV on STS injection as adjunctive therapy vs. Western medicine alone for PHD. STS is sodium tanshinone IIA sulfonate injection and WM is Western medicine

The outcome SV was reported by five RCTs. The results of meta-analysis were shown in Fig. 11 and it could be observed that a moderate heterogeneity (*p* = 0.10, I^2^ = 49%) existed. Hence, the fixed effect model was selected. Results from this meta-analysis (MD = 13.10; 95% CI, 11.83 to 14.38; *p* < 0.001) demonstrated that STS injection as adjunctive therapy displayed better effect in increasing SV than Western medicine alone. As shown in Table S10, robust results were found in the process of sensitivity analysis. Owing to the slight heterogeneity within the included studies, subgroup analysis was conducted basing on STS injection dosage, control group measures and intervention time. Results from subgroup analysis showed that these parameters had no significant interaction, consistently supporting the therapeutic efficacy of STS injection as adjunctive therapy. Publication bias had also been evaluated for the five RCTs and results from Egger’s test (t = -0.64, *p* = 0.57) and Begg’s test (Z = 0.73, *p* = 0.462) suggested that there was no publication bias within the 5 RCTs.

### Risk of bias assessment

**Fig. 12:**
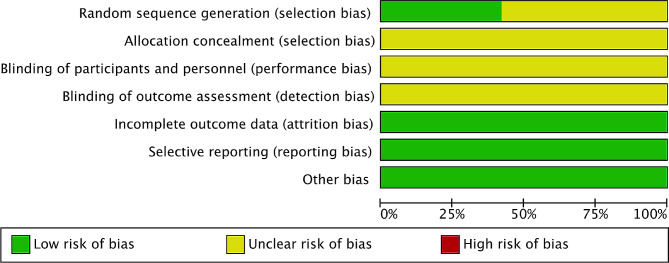
Risk of bias graph: review authors’ judgements about each risk of bias item presented as percentages across all included studies

In Fig. 12, the risk of bias graph of the included 19 RCTs was described. Almost all RCTs reported random sequence generation, but only 8 RCTs reported the specific randomization methods [28–30, 34, 36, 40–42]. All RCTs did not report allocation concealment, blinding of outcome assessment, blinding of participants and personnel, so they were evaluated as “unclear risk”. All outcome data were complete, therefore it was considered that there was no risk of incomplete outcome data. No selective reporting was identified and they were considered as “low risk”. Moreover, none of the included RCTs mentioned any other sources of bias, so our study considered that there was no other bias risks.

### Meta-regression

Meta-regression between the adjunctive therapy of STS injection and using Western medicines alone for PHD in improvement of clinical effective rate, the level of LBV, HBV, PV, HCT, FIB, PaO_2_, PaCO_2_, LVEF and SV based on STS injection dosage, control group measures and intervention time were performed. However, no obvious significant difference was found in the Table S11 (most of p value was more than 0.05).

## Adverse reaction

As shown in Table 4, seven RCTs provided the adverse reactions and other thirteen RCTs did not report. Besides, it could be seen that using Western medicine alone could cause rhinobyon, orthostatic hypotension, tachycardia, dry cough, hypotension, abnormal liver function, urticaria, fever, gastrointestinal reaction, nausea and vomiting for the PHD patients. Similar to the control group, the adverse reactions including rhinobyon, orthostatic hypotension, dry cough, hypotension, abnormal liver function, urticaria, fever, gastrointestinal reaction, nausea and vomiting also existed in the treatment group (STS injection combined with Western medicine). Nevertheless, no serious adverse event was reported in these two groups. In the treatment group, the incidence rate of adverse reaction was 2.31% (21/909), while it was 2.21% (20/906) in the control group. No obvious difference (RR = 1.05; 95% CI, 0.57 to 1.92; Z = 0.15, *p* = 0.88) was observed after analyzing the incidence rates with RevMan 5.4.

**Table 4:** Incidence rate of adverse reaction

Type	Number of adverse reactions		References
	Treatment group	Control group	
Nausea and vomiting	2	3	Chai et al., 2017.
Rhinobyon	1	3	Chai et al., 2017.
Orthostatic hypotension	1	2	Chai et al., 2017.
Tachycardia	0	2	Chai et al., 2017.
Dry cough	7	2	Liang, 2016; Liu et al., 2017; Lu et al., 2014; Mei 2018; Song et al., 2017.
Hypotension	5	1	Liang, 2016; Liu et al., 2017; Lu et al., 2014; Mei 2018; Song et al., 2017.
Abnormal liver function	1	2	Zhang et al., 2018.
Urticaria	2	2	Zhang et al., 2018.
Fever	1	2	Zhang et al., 2018.
Gastrointestinal reaction	1	1	Zhang et al., 2018.
Total event	21	20	
Incidence rate	2.31% (21/909)	2.21% (20/906)	

## Discussion

Previously, some studies on the systematic review or meta-analysis of TCM as adjunctive therapy for PHD were conducted with positive results. Liu et al. conducted a systematic review and meta-analysis involving 35 RCTs with 2715 patients for assessing the efficacy and safety of *salvia miltiorrhiza* injection on PHD [5]. In their results, they found that conventional medicine treatment in combination with *salvia miltiorrhiza* or complex *salvia miltiorrhiza* injection displayed better effects in improving clinical effectiveness rates, PaO_2_ and hemorheology, meanwhile decreasing PaCO_2_ and alleviating mPAP compared with routine medicine treatment alone. Although nice results were concluded by them, several limitations still existed in their work. Firstly, significant heterogeneity was detected among the included RCTs. However, they did not perform the sensitive analysis and subgroup analysis in the study, which could cause an inaccuracy result for the efficacy of *salvia miltiorrhiza* injection. Secondly, only funnel plot was adopted for evaluating publication bias, which could not offer an accuracy result. Thirdly, their work did not follow the PRISMA-guideline, which made the conclusion unreliable. Li et al. conceived another systematic review and meta-analysis for a famous TCM injection (ligustrazine injection) for the treatment of PHD [44]. In their work, they found that ligustrazine injection might be a promising treatment method for PHD since the adjunctive use of ligustrazine injection had better effect in improving the NYHA clinical status (New York Heart Association classification, NYHA) and depression of pulmonary artery hypertension than Western medicine alone. But only four therapeutic indicators were employed for the meta-analysis in their work, which might cause the overestimation or underestimation for ligustrazine injection in the treatment of PHD. Besides, publication bias, sensitive analysis and subgroup analysis also had not been performed by them. Li et al. demonstrated the potential therapeutic effectiveness of shenmai injection combined with conventional medical treatment for PHD [45]. Thirty-three RCTs with 2617 patients were involved in their meta-analysis and it was based on six outcome measures including death, adverse events, NYHA clinical status, hemodynamics, PaO_2_, PaCO_2_. However, as the same reason to the above two studies, sensitive analysis and subgroup analysis were also ignored, which might not be enough to obtain a reliable result on the efficacy of shenmai injection. Many different shortcomings existed in the previous systematic review or meta-analysis for evaluating the adjunctive therapy of TCM for PHD.

PHD is becoming a major problem to menace people’s health and life quality. Recently, many studies showed that STS injection as adjunctive therapy had positive therapeutic effects for PHD. Therefore, this study aimed to demonstrate the efficacy of STS injection as adjunctive therapy for PHD and it was strictly reported according to the PRISMA statement with the items of subgroup analysis, sensitive analysis and publication bias evaluation, which was more comprehensive than previous studies (Table S12). Results showed that STS injection as adjunctive therapy displayed better therapeutic effect than Western medicine alone for the improvement of the clinical effective rate (RR = 1.22; 95% CI, 1.17 to 1.27; *p* < 0.001). Moreover, meta-analysis basing on other outcome measures including PaO_2_, LVEF, SV, LBV, HBV, PV, HCT, FIB and PaCO_2_ also consistently demonstrated the good therapeutic efficacy of STS injection as adjunctive therapy for PHD. Moreover, this meta-analysis showed that the incidence rate of adverse reactions in the control group seemed slightly lower than that of the treatment group. However, results from this study proved that it had no difference between these two groups. Besides, there was no RCT reported serious adverse reactions. On the whole, we could conclude that it was safe when using STS injection for treating PHD.

Previous studies have revealed that STS could decrease mean pulmonary arterial pressure, pulmonary arterial thickness, mean right ventricular pressure, right ventricular hypertrophy index and the right ventricular systolic pressure for hypoxia or monocrotaline induced pulmonary hypertension model in rat. The mechanisms could be attributed to STS in controlling the expression of transient receptor potential canonical proteins, PPAR-γ and Kv2.1 of pulmonary artery. The underlying mechanisms also could be attributed to STS in suppressing Ca^2+^ entry and PKG-PPAR-γ signaling pathway [13, 14]. The definition of PHD is right ventricular failure secondary to pulmonary hypertension and it was caused by pulmonary vascular disease or chronic obstructive pulmonary disease (COPD). Through controlling the signaling pathway of MAPK/HIF-1alpha, STS could reduce many inflammatory cytokines in cigarette smoke-induced COPD mice, such as TNF-α and IL-6 [46, 47]. Moreover, the increase of PaO_2_, LVEF, SV and decrease of LBV, HBV, PV, HCT, FIB and PaCO_2_ in this meta-analysis could be the valid evidence to demonstrate the good efficacy of STS injection as adjunctive therapy in treating PHD.

Although great potentials of STS injection for the treatment of PHD were well demonstrated in this study, several limitations still existed. Firstly, the methodological quality of some RCTs was lower than three scores, indicating the poor quality. Method of randomization was rarely described in some included RCTs, which was the main reason to cause the low-quality RCTs. Hence, to reduce the selection bias, randomization method should be described in the included RCTs. Secondly, in many included RCTs, blinding for investigators or patients had not been provided, which could lead to an inaccurate evaluation for STS injection. Thirdly, the heterogeneity was large and it could be attributed to the inconsistency of dosage, control group measures and intervention time in the included RCTs. Moreover, the current research is not registered and there may be a small deviation, but this study still strictly follow the steps of systematic evaluation (Meta-analysis).

Although the advantages of STS injection as adjunctive therapy for PHD have been well demonstrated in this study, evidence was still lacking owing to the low quality within the included RCTs. Therefore, several recommendations were given to the future RCTs on STS injection for PHD. Firstly, the future designed RCTs should strictly meet with the consolidated standards of reporting trials statement (CONSORT 2010). Secondly, to offer reliable evidences for STS injection, more RCTs with multicentre and high-quality are highly recommended.

## Conclusion

Our study has demonstrated that as adjunctive therapy, STS injection combined with Western medicine seemed to be more effective than Western medicine alone for PHD with available evidences. Nevertheless, owe to the low methodological quality of included RCTs, more RCTs with higher quality were highly recommended to verify the effects of STS injection for PHD.

### Electronic supplementary material

Below is the link to the electronic supplementary material.

Supplementary Material 1

## Data Availability

The data underlying this article are available in the article and its online supplementary materials.

## Abbreviations

CI: Confidence interval
CNKI: China National Knowledge Infrastructure
COPD: Chronic obstructive pulmonary disease
FIB: Fibrinogen
HBV: High shear blood viscosity
LBV: Low shear blood viscosity
LVEF: Left ventricular ejection fraction
MD: Mean difference
PaCO_2_: Partial pressure of carbon dioxide
PaO_2_: Partial pressure of oxygen
PHD: Pulmonary heart disease
PV: Plasma viscosity
RCTs: Randomized controlled trials
RR: Risk ratios
SOCE: Store-operated calcium entry
STS: Sodium tanshinone IIA sulfonate
SV: Stroke volume
TCM: Traditional Chinese medicine
VIP: China Science and Technology Journal Database
WHO: World health organization
